# Quantitative evaluation of metastases in axillary lymph nodes of breast cancer

**DOI:** 10.1038/sj.bjc.6601248

**Published:** 2003-10-28

**Authors:** M Inokuchi, I Ninomiya, K Tsugawa, I Terada, K Miwa

**Affiliations:** 1 Department of Gastroenterologic Surgery, Graduate School of Medical Science, Kanazawa University, 13-1 Takara-machi, Kanazawa 920-8641, Japan

**Keywords:** lymph node metastasis, quantitative RT–PCR, sentinel lymph node, breast cancer

## Abstract

We have established a highly sensitive and quantitative reverse transcriptase–polymerase chain reaction (RT–PCR) method to detect axillary lymph node metastases of breast cancer. Amplifying cytokeratin 19 (CK19) mRNA transcripts using real-time TaqMan PCR made it possible to quantify axillary metastatic burden. Metastases in 358 axillary lymph nodes obtained from 23 breast cancers of 22 patients were investigated by conventional haematoxylin and eosin (H&E) staining, immunohistochemical staining and quantitative RT–PCR assay. The detection rates of axillary lymph node metastasis using H&E staining, immunohistochemistry and RT–PCR assay were 4.5, 5.9 and 13.1%, respectively. RT–PCR assay was the most sensitive of these three methods for detecting lymph node metastases. Cytokeratin 19 mRNA expression values of both histologically and immunohistochemically positive lymph nodes were significantly higher than the values for lymph nodes judged to be negative by both histological and immunohistochemical methods (*P*<0.0001), and those of histologically negative, but immunohistochemically positive lymph nodes were significantly higher than the values for lymph nodes judged to be negative by both histological and immunohistochemical methods (*P*<0.0001). Furthermore, metastatic rates of sentinel nodes were higher than the rates of nonsentinel lymph nodes as measured by all three methods. These results indicate that quantitative RT–PCR assay is a sensitive and reliable method for detecting lymph node metastasis. Furthermore, quantification of metastases in sentinel lymph nodes by quantitative RT–PCR assay may be useful to assess the entire axillary burden of breast cancer patients.

Axillary lymph node status is the most powerful predictor of patient prognosis in cases of breast cancer (Fisher *et al*, 1983), and patients without axillary lymph node metastases have a better prognosis. However, 15–30% of node-negative patients suffer from a recurrence within 5 years (Fisher *et al*, 1983; Giuliano *et al*, 1995; Saimura *et al*, 1999). Previous studies have reported that routine haematoxylin and eosin (H&E) staining analysis of axillary lymph nodes may miss micrometastases in the range of 9–25% of patients that can be readily detected by serial sectioning (International (Ludwig) Breast Cancer Study Group, 1990; Mascarel *et al*, 1992; Nasser *et al*, 1993) and immunohistochemical staining (Mascarel *et al*, 1992; Hainsworth *et al*, 1993; Nasser *et al*, 1993; Cote *et al*, 1999). However, these methods are too cumbersome, too time-consuming, and too costly to be used in routine examinations (Lockett *et al*, 1998).

Several reports have indicated that the detection of micrometastases using reverse transcriptase–polymerase chain reaction (RT–PCR) assay may be clinically important (Noguchi *et al*, 1994,^1996^; Schoenheld *et al*, 1994; Mori *et al*, 1995,^1998^; Masuda *et al*, 2000; Manzotti *et al*, 2001). Moreover, screening for metastatic disease in axillary lymph nodes by RT–PCR amplification has been shown to be more sensitive and cost effective than serial sectioning and immunohistochemical staining (Lockett *et al*, 1998). However, there are also reports on the limitations of this method (Zippelius *et al*, 1997; Bostick *et al*, 1998a,1998b; Ko *et al*, 2000). The major limitation is related to the specificity in the detection of metastatic cancer cells. The target genes can even be expressed in blood samples from healthy humans and noncancerous patients. This is partially attributable to the nonquantitativeness of the conventional RT–PCR assay, in which PCR products are visualised by staining with ethidium bromide or autoradiograms, and for which detection depends on the number of amplification cycles and the design of the primers. The expression of the target gene has been defined only as positive or negative and not quantified in conventional RT–PCR assay. The detection of lymph node metastases would be more reliable by quantitative evaluation of PCR products, because faint signals from normal lymphocytes could be distinguished from cancer-related signals by the difference in signal intensities.

Recently, a real-time detection and quantitative PCR assay has been developed, which is based on the TaqMan methodology (Gibson *et al*, 1996). Its advantage is the specific detection of rare events. The sensitivity of the assay allows for the detection of 10–100 pg of RNA. This method uses the 5′–3′ exonuclease activity of *Taq* polymerase to cleave a dual-labelled probe annealed to a target sequence during the extension phase of PCR. It is highly reproducible and quantitative, and it eliminates the major risks of contamination encountered with other types of detection (nested PCR and competitive PCR). In addition, no post-PCR manipulations are required with this method, and quantification and calculation of the results are all automated.

Currently, many investigators reported sentinel lymph node biopsy for the diagnosis of axillary lymph node metastases from breast cancer (Giuliano *et al*, 1994; Albertini *et al*, 1996; Veronesi *et al*, 1997; Krag *et al*, 1998). The sentinel lymph node is the first lymph node to receive drainage from the primary tumours in early stage cancers (Morton *et al*, 1992). Therefore, a tumour-free sentinel lymph node implies the absence of lymph node metastases in the entire lymphatic basin. This sentinel node concept had been previously confirmed by H&E examinations, immunohistochemical study and nonquantitative RT–PCR assays (Giuliano *et al*, 1994; Albertini *et al*, 1996; Veronesi *et al*, 1997; Krag *et al*, 1998; Kataoka *et al*, 2000; Manzotti *et al*, 2001; Branagan *et al*, 2002; Ishida *et al*, 2002).

The purpose of this study was to develop a quantitative RT–PCR assay based on real-time TaqMan PCR for detecting and quantifying metastases in axillary lymph nodes of breast cancer, and also to study the feasibility of the sentinel lymph node biopsy technique by using quantitative RT–PCR assay.

## MATERIALS AND METHODS

### Patients and treatment (sentinel lymph node biopsy)

A total of 22 Twenty-two women with a case of Tis (carcinoma *in situ*), clinical stage I or II primary breast cancers (one with bilateral breast cancers), were treated by the Department of Surgery , Kanazawa University Hospital between June 1999 and September 2000. Two cases had already undergone excisional biopsy at the other hospital. After receiving informed consent, the patients were scheduled to undergo preoperative lymphoscintigraphy and sentinel lymph node biopsy. The protocol was approved by the Local Ethics Committee at the Kanazawa University School of Medicine. Lymphatic mapping and sentinel lymph node biopsy were performed as described previously (Tsugawa *et al*, 2000). Then the primary lesion was removed. Finally, a complete dissection (level I–II) of the axillary lymph nodes was performed conventionally. The operative methods included modified radical mastectomy in 16 patients (one received bilateral mastectomy) and wide excision with axillary lymph node dissection in six patients. The clinicopathological findings were classified according to the Histologic Classification of Breast Cancer proposed by the Japanese Breast Cancer Society (The Japanese Breast Cancer Society, 2000), which are modified histological types from the World Health Organization.

### Clinical samples

#### Lymph node samples

After sentinel lymph node biopsy, each lymph node was entirely cut into 2 mm thick slices. Each node was cut into two to 10 slices. For each sliced node, even number of these slices was examined by routine H&E and immunohistochemical staining as permanent sections. The odd number of these slices was subjected to RT–PCR assay.

**Figure 1:**
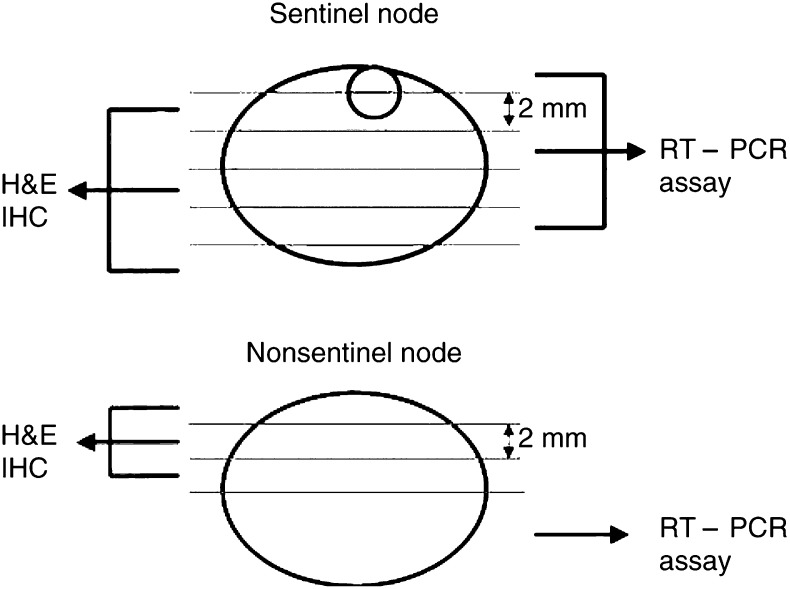
Section of the lymph node samples.

After axillary lymph node dissection, the lymph nodes were macroscopically isolated from the specimens. Each node was cut into halves. One half being examined by H&E and immunochemical staining was cut into more 2 mm thick slices (one to four sections); and the remaining half being subjected to RNA extraction (Figure 1

).

#### Cancer cell line and normal control

The human mammary cancer cell line MCF-7 was used as a positive control for cancer cell detection by quantitative RT–PCR assay. The cell line was maintained in RPMI 1640 (Nissui Pharma. Co., Ltd, Tokyo, Japan) supplemented with 10% heated, inactivated fetal bovine serum (Life Technologies, Inc., Gaithersburg, MD, USA) at 37°C and 5% CO_2_.

As a negative control, 24 normal lymph nodes from non-cancerous patients (chronic cholecystitis and haemorrhagic gastric ulcer) were obtained at the time of surgery (cholecystectomy or gastrectomy) under the informed consents and studied in order to determine the cutoff value of quantitative RT–PCR assay.

### Total RNA extraction and first-strand cDNA synthesis

The acid guanidinium thiocyanate–phenol–chloroform extraction procedure was used for the extraction of total RNA from MCF-7, primary carcinoma tissue, corresponding normal breast tissue, dissected lymph nodes, peripheral mononuclear cells from healthy volunteer and normal lymph nodes (Chomczynski and Sacchi, 1987). The quantity and quality of the extracted RNA was confirmed by absorption measurement at 260 and 280 nm using the spectrophotometer (U-2000A, HITACHI, Tokyo, Japan). The prepared RNA (10 *μ*g) was mixed with 500 pmol of the oligo dT primer for 15 min at 68°C. After heat-denaturing, each sample was quickly chilled on ice for 5 min. The RNA sample was reverse-transcribed at 42°C for 60 min into first-strand cDNA in reverse-transcription solution (400 U of Moloney murine leukaemia virus reverse transcriptase (Life Technologies, Inc.), 50 mM Tris-HCl (pH 8.3), 75 mM KCl, 3 mM MgCl_2_, 0.01 M DTT, 0.5 mM each dNTP, and 16 U of RNasin (Promega, Madison, WI, USA) with a total volume of 100 *μ*l.

### Quantitative PCR assay

We focused on cytokeratin 19 (CK19) as RNA targets to detect cancer cells in the axillary lymph node. Optimal primers and probes were selected using Software Primer Express Ver.1.7 provided by PE Applied Biosystems (Foster, CA, USA). The TaqMan glyceraldehyde-3-phosphate dehydrogenase (GAPDH) Control Reagent (PE Applied Biosystems) was used for PCR of GAPDH mRNA as an internal reference. The sequences of primers and internal probes for CK19 and GAPDH mRNA are given in Table 1

**Table 1:** Primer and probe sequences for quantitative RT–PCR

**Target gene**	**Sequence 5′–3′**
CK19	Forward primer GAAGAACCATGAGGAGGAAATCA
	Reverse primer ACCTCATATTGGCTTCGCATGT
	Probe (FAM)-CGGGCACCGATCTCGCCAAG-(TAMRA)
GAPDH	Forward primer GAAGGTGAAGGTCGGAGTC
	Reverse primer GAAGATGGTGATGGGATTTC
	Probe (JOE)-CAAGCTTCCCGTTCTCAGCC-(TAMRA)

CK19=cytokeratin 19; GAPDH=glyceraldehyde-3-phosphate dehydrogenase; FAM=6-carboxy-fluorescein, reporter dye; JOE=2,7-dimethoxy-4,5-dichloro-6-carboxy-fluorescein), reporter dye; TAMRA=6-carboxy-tetramethyl-rhodamine, quencher dye.

.

In order to prevent amplification from the processed pseudogene (CK19a and CK19b) (Ruud *et al*, 1999), primers and internal probes were designed to maximise the sequence difference between CK19 and its pseudogenes. The internal probe for CK19 was labelled with a reporter dye (FAM: 6-carboxy-fluorescein) at the 5′-end and a quencher dye (TAMRA: 6-carboxy-tetramethyl-rhodamine) at the 3′-end.

The PCR solution (50 *μ*l) was composed of 1 *μ*l of cDNA solution corresponding to 100 ng of total RNA, 200 nM of each of the forward and reverse primer, 100 nM of internal probe and TaqMan Universal Master Mix (PE Applied Biosystems), which contained Ampli-Taq Gold DNA polymerase, a reaction buffer, dNTP, dUTP and AmpErase uracil-*N*-glycosylase. PCR was carried out for 2 min at 50°C, 10 min at 95°C followed by 40 cycles of 15 s at 95°C and 1 min at 60°C using the ABI 7700 Prism Sequence Detector (PE Applied Biosystems). PCR amplification of GAPDH mRNA was conducted according to the manufacturer's instructions for the TaqMan GAPDH Control Reagent.

The quantification of mRNA levels of the target gene was made using real-time fluorescence detection ‘TaqMan TM’ technology.

Reactions were characterised at the point during cycling when amplification of the PCR product is first detected, rather than according to the amount of PCR product accumulated after a fixed number of cycles. The larger the starting quantity of the target molecule, the earlier a significant increase in fluorescence was observed. The parameter threshold cycle (Ct) is defined as the fractional cycle number at which the fluorescence generated by cleavage of the probe passes a fixed threshold above baseline. The target message in unknown samples is quantified by measuring Ct and by using a standard curve to determine the starting target message quantity. Standard curves for target genes were generated using serial dilutions (10^−1^–10^−5^) of RNA derived from breast cancer cell line (MCF-7 cells) in each assay.

Both the precise amount and quality of total RNA added to each reaction mix (based on absorbance) are difficult to assess. Therefore, we also quantified transcripts of the GAPDH gene as an internal reference according to a quantitative PCR assay, and each sample was normalised on the basis of its GAPDH content. Lymph node samples were considered eligible for study when the GAPDH Ct value was ⩽35, that is, suggesting an appropriate starting amount and quality of total RNA.

The amounts of target mRNA in samples were calculated relative to those in MCF-7 and standardised according to those of GAPDH mRNA. The quantification value of CK19 mRNA was described as an amount relative to the MCF-7.

### Immunohistochemistry

To confirm the quantitative RT–PCR analysis histologically, an immunohistochemical examination was performed for all the lymph nodes. Anti-cytokeratin 19 antibodies (DAKO, Glostrup, Denmark) were used for detecting metastatic cancer cells in the lymph nodes. Immunohistochemistry was carried out by using the Envision+ peroxidase kit (DAKO). Lymph nodes were scored positive when the cells stained clearly CK19 and had the morphological characteristics of malignant cells.

### Statistical analysis

Categorical variables were compared using the *χ*^2^ test, employing the Yates correction when necessary. Continuous variables were compared using the Mann–Whitney *U*-test. All *P*-values were two-sided. A *P*-value of less than 0.05 indicated a significant difference.

## RESULTS

### Study I: Quantification of metastases in axillary lymph nodes

#### Characteristics of patients and tumours

A total of 358 axillary lymph nodes were obtained from 23 breast cancers of 22 patients. The general characteristics of the patients with breast cancer are presented in Table 2

**Table 2:** Clinicopathological characteristics of patients with breast cancer

**Age (mean) (years)**	**30–77 (55)**
*Menopausal status*	
Pre	9
Post	13
*Pathological stage (TNM)*	
0	1
I	7
IIA/B	14
IIIA	1
*Histological type of tumour*	
Invasive ductal cancer	21
Noninvasive ductal cancer	2
*Lymphatic invasion*	
Positive	13
Negative	8
Unknown^a^	2
*Axillary involvement (histology)*	
Present	6
Absent	17
*Operation methods*	
Modified radical mastectomy	17
Breast-conserving surgery	6

a After excisional biopsy state.

. The average age of the patients was 53 years (range 30–77). In all, 10 patients had clinically negative nodes (N0) (one with bilateral N0), and 12 had positive nodes (N1).

#### Haematoxylin and eosin staining and immunohistochemistry

**Figure 2:**
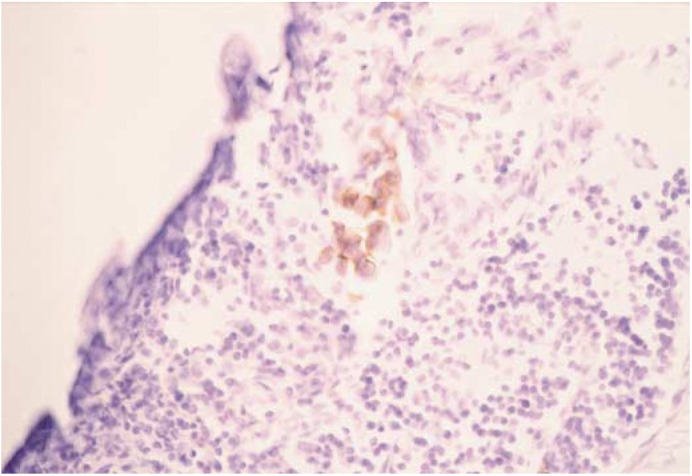
Immunohistochestochemical detection of cancer cells in the axillary lymph node. Photomicrograph of CK19-positive cancer cells in lymph node (magnification × 400).

Among 358 lymph nodes from 23 tumours, 16 nodes (4.5%) in six cases had tumour involvement as indicated by staining of H&E. All the histologically positive lymph nodes indicated by H&E also had CK19-expressing tumour cells indicated by immunohistochemical methods. Moreover, five of the histologically negative lymph nodes indicated by H&E had occult metastases indicated only by immunohistochemical methods (Figure 2

). In total, 21 nodes (5.9%) in nine cases had lymph node metastases indicated by immunohistochemistry.

#### Sensitivity of quantitative RT–PCR assay

**Figure 3:**
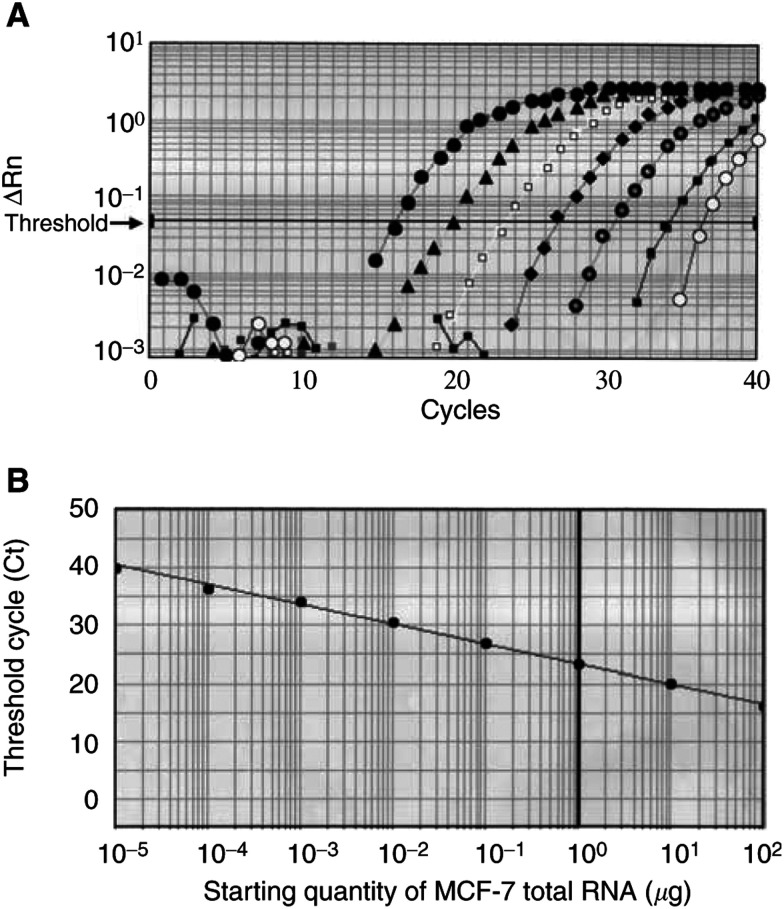
Amplification of CK19 mRNA by quantitative PCR assay. (**A**) Amplification plots of CK19 mRNA for serial dilution of MCF-7 total RNA. ΔRn is defined as the cycle-to-cycle change in the reporter fluorescence signal normalised to a passive reference fluorescence signal. The initial amount of total RNA is displayed: •, 10^2^ ng; ▴, 10 ng; □, 1 ng; ♦, 10^−1^ ng; 
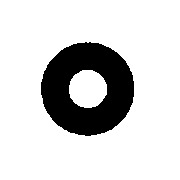
, 10^−2^ ng; ▪, 10^−3^ ng and ○, 10^−4^ ng. Ct is calculated as the cycle at which fluorescence signal passes a fixed threshold line. (**B**) Standard curve of the CK19 RT–PCR assay. Ct is plotted against the starting quantity of MCF-7 total RNA.

In order to estimate the sensitivity of RT–PCR assays, serial dilution experiments were carried out. A volume of 100 ng of RNA derived from MCF-7 was diluted from 10^−1^ to 10^−6^ and subjected to RT–PCR assay. In Figure 3A

, amplification plots for the sample containing serial dilutions of RNA derived from MCF-7 cells are overlaid. The standard curve was constructed from plots for the calculated Ct values of each reaction (Figure 3B). Figure 3B shows that it is possible to quantify CK19 mRNA expression in spans of five logs. CK19 transcripts were detected up to 10^−4^ corresponding to 10^−6^ *μ*g of MCF-7 total RNA. Cytokeratin 19 assays of MCF-7 cell-derived genomic DNA results do not arise from amplification of the processed pseudogenes. The same results were obtained for GAPDH mRNA (data not shown).

#### Quantification of CK19 mRNA expression in lymph nodes

**Figure 4:**
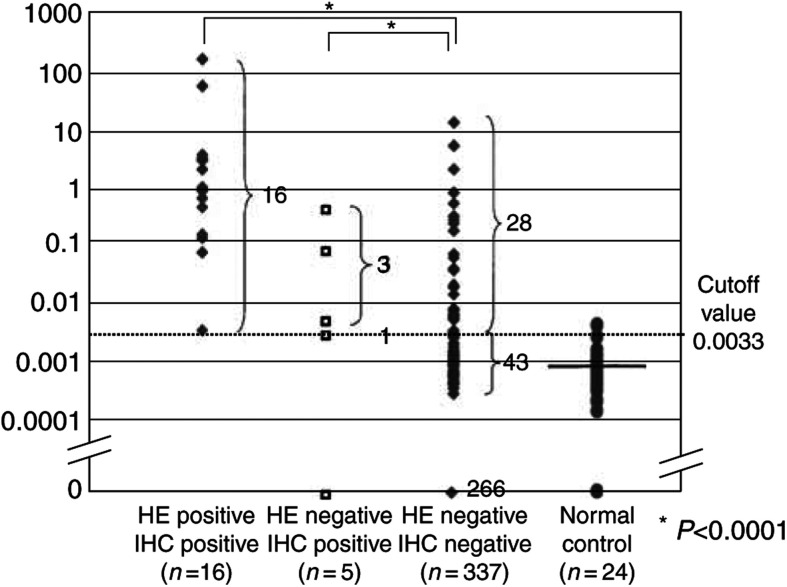
Relative expression of CK19 mRNA.

Among 358 lymph nodes, 91 (25.4%) showed CK19 mRNA expression (2.8 × 10^−4^–1.7 × 10^2^) (Figure 4

). All the 16 histologically positive lymph nodes showed CK19 mRNA expression. Four nodes showed CK19 mRNA expression among five nodes, in which lymph node metastasis was detected only by immunohistochemistry. The expression values of both histologically and immunohistochemically positive lymph nodes ranged from 2.5 × 10^−3^ to 1.7 × 10^2^, which were significantly higher than the values for either histologically or immunohistochemically determined negative lymph nodes, for which values ranged from 0 to 1.4 × 10 (*P*<0.0001). Furthermore, the expression values of histologically negative, but immunohistochemically positive lymph nodes ranged from 0 to 5.3 × 10^−1^, which were significantly higher than both the values for either histologically or immunohistochemically determined negative nodes (*P*<0.0001).

In order to exclude false positives, we used as the cutoff value the average mRNA value+2 s.d. of CK19 mRNA expression in normal lymph nodes from noncancerous patients (3.3 × 10^−3^ for CK19). Values above or equal to this cutoff value of CK19 mRNA were defined as CK19 mRNA positive. The results of the comparison between histological examination and quantitative RT–PCR method using a cutoff value are shown in Table 3

**Table 3:** Relation between histological examination and quantitative RT–PCR method in the detection of metastasis in 358 axillary lymph nodes

	**Histological analysis**	
**RT–PCR analysis (CK19)**	**Negative (%) (*n*=342)**	**Positive (%) (*n*=16)**
Negative	311 (90.9)	0
Positive	31 (9.1)	16 (100)

CK19=cytokeratin 19.

. Using the cutoff value method, 31 (9.1%) of histologically negative lymph nodes were determined to be CK19 positive (Table 3). Moreover, three out of four of the nodes that were determined to be histologically negative, but immunohistochemically positive, were determined to be CK19 mRNA positive. Thus, CK19 mRNA positive nodes were strongly expected to possess cancer cells in the lymph nodes. Therefore, the cutoff value of CK19 mRNA expression was used as described above in the subsequent study.

In the detection of breast cancer metastases in axillary lymph nodes, immunohistochemistry and the RT–PCR method were more sensitive than the usual H&E methods in terms of their ability to detect axillary lymph node metastasis (Table 4

**Table 4:** Detection of metastasis by H&E, IHC and the RT–PCR method on 358 axillary lymph nodes

**Detection procedure**	**Metastatic ratio (%)**	
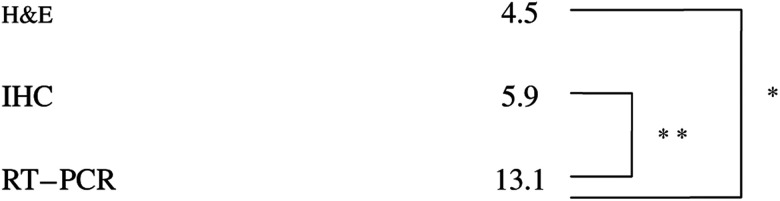		

H&E=haematoxylin and eosin; IHC=immunohistochemistry

^*^P<0.0001,

^**^P<0.005.

). Furthermore, metastatic rates of axillary lymph nodes using immunohistochemistry and the RT–PCR method were higher than the rates observed when using the H&E methods (Table 5

**Table 5:** Axillary lymph node metastasis detection by histological examination (HE), immunohistochemistry (IHC) and quantitative RT–PCR method in 22 breast cancer patients

	**Axillary lymph node metastasis**		
**Detection procedure**	**Positive**	**Negative**	**Metastatic rate (%)**
HE	6	17	26
IHC	9	14	39
RT–PCR	15	8	65

).

### Study II: Metastasis detection in sentinel lymph nodes

#### Identification of sentinel lymph node

Intraoperative sentinel lymph node identification of axillary lesions was successful in all cases (100%). Each sentinel lymph node mapping detected one to four (mean 2.0) lymph nodes. In total, 45 sentinel lymph nodes were identified and 358 lymph nodes, including sentinel nodes, were dissected. The axillary sentinel lymph nodes contained cancer metastases in six (26%) of 23 breasts in 22 patients by H&E staining. For two of the patients, only the sentinel lymph nodes were metastatic nodes, whereas the remaining four breasts also had metastasis in other axillary nodes. The evaluation of metastasis by conventional H&E staining revealed that this sentinel node biopsy technique is 100% in terms of both diagnostic accuracy and sensitivity.

#### Quantification of CK19 mRNA expression of sentinel lymph nodes and nonsentinel lymph nodes

No significant differences of CK19 mRNA expression values were found between sentinel lymph nodes and nonsentinel lymph nodes. Among 39 of the histologically negative sentinel lymph nodes, CK19 mRNA positive nodes were five (12.8%). Two of the five nodes had immunohistochemically CK19 positive metastases. However, two nodes showing immunohistochemically CK19 positive cancer cluster did not show CK19 mRNA signal by quantitative RT–PCR assay among histologically negative sentinel lymph nodes. In total, six of the tumour-involved sentinel lymph nodes were detected by H&E staining and seven more tumour-involved sentinel lymph nodes were detected using either immunohistochemistry or RT–PCR assay.

The metastatic rate for the sentinel lymph nodes was significantly higher than that of the nonsentinel nodes, irrespective of which of the three methods was used. The metastatic rates of the sentinel and nonsentinel lymph nodes as indicated by the RT–PCR method were higher than those resulting from H&E and immunohistochemical staining (Table 6

**Table 6:** Detection of axillary sentinel lymph node (SLN) metastasis by various procedures

	**Metastasis Number (%)**		
	**SLN**	**non-SLN**	
**Detection procedure**	***n*=45**	***n*=304**	***P*-value**
H&E	6 (13.3)	10 (3.2)	<0.001
IHC	10 (22.2)	11 (3.5)	<0.0001
RT–PCR	11 (24.4)	36 (11.8)	<0.05

H&E=haematoxylin and eosin; IHC=immunohistochemistry.

).

## DISCUSSION

In this study, we have established a highly sensitive and quantitative RT–PCR assay to detect axillary lymph node metastases of breast cancer. We also proved the reliability of our sentinel lymph node biopsy technique by using this RT–PCR assay. Furthermore, we could assess the quantity of metastatic cancer cells in the axillary lymph nodes.

Usually, lymph node metastasis is assessed only in one section from the centre of the lymph node using H&E or immunohistochemical staining. These examination methods carry the risk of ignoring the metastatic foci if they are present at the periphery of the node. The RT–PCR assay offers the advantage of metastasis detection in the whole node as compared to H&E and immunohistochemical staining.

Recently, RT–PCR assays with tumour-specific gene markers such as CK19, CEA, MUC1, PIP, Mapsin and mammaglobin B have been widely used to detect occult metastasis in cancer patients (Noguchi *et al*, 1994,^1996^; Schoenheld *et al*, 1994; Mori *et al*, 1995,^1998^; Kataoka *et al*, 2000; Masuda *et al*, 2000; Manzotti *et al*, 2001; Mitas *et al*, 2001). To date, however, no marker that is expressed in all breast cancer tissues, but not in normal lymph node has been available, partly because of the heterogeneity of marker gene expression in cancer cells. Therefore, multimarker analysis will probably be necessary for the precise and accurate examination of lymph node metastasis (Manzotti *et al*, 2001; Mitas *et al*, 2001). On the other hand, some evidence suggests that false positives may arise due to several factors, including illegitimate CK19 mRNA expression in lymphocytes, the presence of noncancerous epithelial cells in lymph nodes and the use of inappropriate primer modification and too many PCR amplification cycles (Bostic *et al*, 1998b). In the present study, we designed our original CK19 primers and probes to minimise amplifying illegitimate mRNA. Using the ABI 7700 Prism Sequence Detector (PE Applied Biosystems) and our original primers and probes, we could quantify the target gene, and minimise amplifying inappropriate genes. We also amplified and quantified CEA and mammaglobin B mRNA as multimarkers for metastasis detection (data not shown). The detection of metastasis by mammaglobin B resulted in low sensitivity. The signal intensity of CEA mRNA from normal lymph nodes was extremely high. Therefore, we selected CK19 as the target gene in this study.

It is unclear whether the signal showed by RT–PCR assay reflects cancer-specific signals. It was not possible to show the RT–PCR positive cancer cells histologically. In the present study, we extracted RNA from one entire half of a lymph node, and the remaining half was examined using H&E or immunohistochemical staining. Histologically positive lymph nodes showed significantly higher CK19 mRNA values, while histologically negative lymph nodes showed significantly lower rates. Surprisingly, most of the histologically negative, but immunohistochemically positive, lymph nodes showed high CK19 mRNA value. These results indicated that cancer detection using quantitative RT–PCR assay is reliable. Although the result of immunohistochemistry and RT–PCR was not necessarily in agreement in our study, this is probably due to the fact that the same portions of the lymph node samples were not examined by both methods. The rate of lymph node metastatic detection goes up by increasing the volume of the lymph nodes examined, and increases even further by combining RT–PCR and immunohistochemistry.

The detection of metastases would be more reliable if quantitative evaluation were employed in the RT–PCR assay, because faint signals from normal lymphocytes could be distinguished from cancer-related signals by the signal intensity difference. In order to eliminate false positives resulting from amplification of the illegitimate CK19 expression from lymph nodes, we attempted to determine the cutoff value that could distinguish the cancer-specific expression from illegitimate expression, and selected a more sensitive and reliable one. Although the cutoff value that we selected was set at a relatively high value, our quantitative RT–PCR assay was sensitive enough. It is speculated that the higher CK19 mRNA levels might reflect a higher tumour burden in the lymph node. However, it is still unclear for just how few a number of cancer cells it would be possible to only detect by RT–PCR assay and still be capable of growing to form clinically detectable metastatic foci.

A sentinel lymph node is defined as the first lymph node that receives the lymphatic drainage from the primary tumour to reach the lymph node. We previously reported the usefulness of sentinel lymph node biopsy using a dual mapping procedure combining dye-guided and gamma probe-guided methods, and demonstrated a high diagnostic accuracy of 95%; a sensitivity of 89%, and a specificity of 100% were achieved in the diagnosis of axillary metastasis (Tsugawa *et al*, 2000). Giuliano *et al* (1994) reported that sentinel lymph nodes were significantly more likely to contain metastases than nonsentinel lymph nodes removed during axillary lymph node dissection. Our study also demonstrated that sentinel lymph nodes contain metastases more frequently than nonsentinel lymph nodes irrespective of metastasis detection procedure. These results may partly depend upon the different sampling procedures between sentinel and nonsentinel nodes by H&E or immunohistochemistry, as Figure 1 was described. However, the whole half volume of the entire lymph nodes either in sentinel or nonsentinel nodes was examined by RT–PCR assay. Therefore, in the RT–PCR assay, we could justifiably compare the metastatic rate in sentinel node with nonsentinel node regardless of the extent of the sampling procedure. We could confirm the reliability of the sentinel lymph node concept using the quantitative RT–PCR technique to detect metastasic cancer cells. RT–PCR of only sentinel nodes based on the sentinel node hypothesis would save time and reduce cost to detect axillary lymph node metastasis in breast cancer patient.

It remains controversial whether micrometastatic disease in pathology-negative, but RT–PCR-positive, lymph nodes is clinically significant. Several studies report clinical relevance of RT–PCR for the detection of axillary lymph node metastasis in breast cancer (Masuda *et al*, 2000; Sakaguchi *et al*, 2003). However, these studies had used conventional RT–PCR assay and was relatively subjective. Since the results obtained from conventional RT–PCR assay may change under various experimental conditions, it is difficult to compare accurately the amount of metastatic cancer cell in lymph node with the patient prognosis. Godfrey *et al* (2001) reported that quantitative RT–PCR in oesophageal cancer offers significant benefits over standard RT–PCR and identifies node-negative patients at high risk for recurrence. The application of quantitative RT–PCR assay in lymph node of breast cancer patients might disclose the prognostic significance of micrometastatic disease. In the present study, we could not assess the prognostic value of micrometastatic disease or the amount of metastatic cells using quantitative RT–PCR assay, because of the small sample size and the short observation time. However, in the future, the clinical implication of quantitative RT–PCR findings will be derived from the clinical follow-up of these patients or a more large-scale study.

Although it is well established that axillary lymph node status is the most powerful predictor of patient outcome in breast cancer, no study had focused on the quantity of cancer cells present in the metastatic lymph nodes, because it was considered up to now that no method could evaluate objectively the quantity of metastatic cancer cells. On the other hand, it is cumbersome to quantify cancer cells in all resected lymph node specimens. As described above, if the sentinel lymph nodes can be used to predict axillary lymph node status, the quantity of cancer cells in axillary lymph node specimens can be replaced by focusing on the quantification in the sentinel lymph node specimens. Within this framework, the application of our method to sentinel lymph node biopsy may be a useful prognostic factor. It will be necessary to ascertain whether the quantitative value of cancer cells in the sentinel lymph nodes really does exhibit some relationship to the prognosis and the need for additional, adjuvant therapy by following up further with these patients.
